# Evaluation of Large Language Model Performance in Answering Clinical Questions on Periodontal Furcation Defect Management

**DOI:** 10.3390/dj13060271

**Published:** 2025-06-18

**Authors:** Georgios S. Chatzopoulos, Vasiliki P. Koidou, Lazaros Tsalikis, Eleftherios G. Kaklamanos

**Affiliations:** 1 Department of Preventive Dentistry, Periodontology and Implant Biology, School of Dentistry, Aristotle University of Thessaloniki, 54124 Thessaloniki, Greece; tsalikis@dent.auth.gr (L.T.); ekaklam@dent.auth.gr (E.G.K.); 2 Division of Periodontology, Department of Developmental and Surgical Sciences, School of Dentistry, University of Minnesota, Minneapolis, MN 55455, USA; 3 Centre for Oral Immunobiology and Regenerative Medicine and Centre for Oral Clinical Research, Institute of Dentistry, Queen Mary University London (QMUL), London E1 4NS, UK; v.koidou@qmul.ac.uk; 4 School of Dentistry, European University Cyprus, Nicosia 2404, Cyprus; 5 Hamdan bin Mohammed College of Dental Medicine, Mohammed bin Rashid University of Medicine and Health Sciences (MBRU), Dubai P.O. Box 505055, United Arab Emirates

**Keywords:** artificial intelligence, ChatGPT, Google Gemini, Microsoft Copilot, periodontics, furcation

## Abstract

**Background/Objectives**: Large Language Models (LLMs) are artificial intelligence (AI) systems with the capacity to process vast amounts of text and generate human-like language, offering the potential for improved information retrieval in healthcare. This study aimed to assess and compare the evidence-based potential of answers provided by four LLMs to common clinical questions concerning the management and treatment of periodontal furcation defects. **Methods**: Four LLMs—ChatGPT 4.0, Google Gemini, Google Gemini Advanced, and Microsoft Copilot—were used to answer ten clinical questions related to periodontal furcation defects. The LLM-generated responses were compared against a “gold standard” derived from the European Federation of Periodontology (EFP) S3 guidelines and recent systematic reviews. Two board-certified periodontists independently evaluated the answers for comprehensiveness, scientific accuracy, clarity, and relevance using a predefined rubric and a scoring system of 0–10. **Results**: The study found variability in LLM performance across the evaluation criteria. Google Gemini Advanced generally achieved the highest average scores, particularly in comprehensiveness and clarity, while Google Gemini and Microsoft Copilot tended to score lower, especially in relevance. However, the Kruskal–Wallis test revealed no statistically significant differences in the overall average scores among the LLMs. Evaluator agreement and intra-evaluator reliability were high. **Conclusions**: While LLMs demonstrate the potential to answer clinical questions related to furcation defect management, their performance varies. LLMs showed different comprehensiveness, scientific accuracy, clarity, and relevance degrees. Dental professionals should be aware of LLMs’ capabilities and limitations when seeking clinical information.

## 1. Introduction

The rise in artificial intelligence (AI) has captured significant attention across numerous fields in recent years. A notable development within AI is Large Language Models (LLMs), which are designed to process vast quantities of text and generate coherent, human-like language [1]. These models offer the potential for faster and more accessible information retrieval compared to traditional methods. LLMs present significant opportunities to revolutionize healthcare. They can enhance efficiency and accuracy by automating tasks such as extracting data from electronic health records, summarizing and structuring medical information, clarifying complex medical texts, and optimizing administrative workflows in clinical settings [2,3,4]. Furthermore, LLMs can contribute to advancements in medical research, quality assurance, and educational initiatives [2,3,4]. Emerging evidence also suggests their potential in diagnostic support and prognostic modeling [5]. Deep learning algorithms, a subset of AI, demonstrate remarkable capabilities in analyzing medical images for disease detection and personalizing treatment plans, often achieving greater accuracy and efficiency than human capabilities [6]. Furthermore, AI holds promise in identifying individuals at risk of specific diseases, enabling tailored preventative and treatment strategies [7].

In dentistry, AI is emerging as a valuable tool for dental professionals, aiding in diagnosis, treatment planning, image analysis, and the prediction of treatment outcomes, and streamlining record-keeping and workflow [8]. Deep learning models show potential in the early detection of dental issues like caries and periapical periodontitis, leading to improved treatment decisions and patient care while saving clinical time [9,10]. Beyond enhancing professional capabilities, these LLM applications foster better-informed decision-making and superior patient outcomes in dentistry. While healthcare providers increasingly use LLMs for quick access to information from diverse sources, concerns regarding the accuracy and reliability of their responses, potential biases, and ethical and legal implications have been raised [11,12]. Studies evaluating the performance of LLMs in answering medical questions have revealed varying levels of accuracy, possibly influenced by inconsistencies in study design and reporting [13].

The integration of LLMs into everyday clinical workflows faces significant challenges. A key issue is the lack of transparency and accessibility regarding the data underpinning LLM-generated answers. Studies also indicate a tendency for LLMs to “hallucinate” or produce misinformation when faced with knowledge gaps [14]. Moreover, the prevalence of digital barriers requiring users to pay a fee or subscribe to view the full content restricts the availability of scientific information needed for training models like ChatGPT [15]. Compounding this, LLMs often have fixed information cut-off dates (September 2021 for GPT-4), which limits their capacity to synthesize responses using the most current scientific literature [16].

Clinicians face significant challenges in successfully treating molars with Class II and III furcation defects, exhibiting a higher rate of tooth loss [17,18]. The complex topography of furcation defects hinders proper debridement, posing the main clinical challenge for their treatment [19,20,21]. Non-surgical treatment has shown limited success, and a systematic review indicated that surgical debridement only provides modest improvement in clinical parameters [22,23,24]. A recent meta-analysis aimed to compare the clinical effectiveness of regenerative periodontal surgery versus open flap debridement for treating furcation defects and to evaluate different regenerative approaches [25]. The analysis of 20 randomized controlled trials revealed that regenerative techniques significantly outperformed open flap debridement regarding furcation improvement, horizontal and vertical clinical attachment level gain, and probing pocket depth reduction. Specifically, treatments involving bone replacement grafts demonstrated the highest probability of achieving the best outcomes for horizontal bone level gain. In contrast, non-resorbable membranes combined with bone replacement grafts ranked highest for vertical attachment level gain and pocket depth reduction [25].

The ability of chatbots to accurately respond to clinically essential questions has been assessed across various dental specialties, including pediatric dentistry [26], operative dentistry [27], oral and maxillofacial radiology [28], orthodontics [29], public health dentistry [30], endodontics [31], prosthodontics [32], oral pathology [33], dental trauma [34], periodontology [35], and implant dentistry [36]. A preliminary study explored how AI-powered face enhancement, specifically using FaceApp, could assist orthodontists in treatment planning by modifying facial attractiveness, and the authors indicated that AI-enhanced images were consistently rated as more attractive by respondents, with significant changes observed in features like lip fullness, eye size, and lower face height, suggesting a potential for AI to guide soft-tissue-focused and personalized orthodontic treatments [37]. As LLMs increasingly become a resource for dental information, evaluating their performance in this field is crucial. Considering the diverse approaches to treating furcation defects and the clinical importance of managing teeth with furcation involvement, it is particularly important to determine if AI can provide accurate and reliable answers. This research aimed to assess and compare the ability of four LLMs to offer evidence-based answers to typical clinical inquiries concerning the management and treatment of periodontal furcation defects. The study’s null hypothesis proposed no significant differences in the comprehensiveness, scientific accuracy, clarity, and relevance of the LLMs’ responses compared to the accepted scientific evidence and guidelines for addressing furcation defects.

## 2. Materials and Methods

This study investigated the performance of four popular LLMs in providing evidence-based answers to clinical questions specifically related to the treatment and management of periodontal furcation defects. As part of a larger set of ten periodontal questions (Table 1) developed by the EFP’s S3 guidelines for periodontitis [38], these furcation-focused inquiries aimed to reflect common scenarios encountered by general dentists. The LLM-generated responses were rigorously compared against a “gold standard” derived from EFP guidelines [38] and relevant recent systematic reviews [39,40,41,42,43,44,45,46] on furcation defect management and treatment. The evaluated LLMs were ChatGPT (GPT 4.0), Google Gemini (Gemini 2.0 Flash Experimental), Google Gemini Advanced (powered by Gemini Ultra 1.0), and Microsoft Copilot (Free Version).

Two American Board of Periodontology-certified periodontists (G.S.C. and V.P.K.) independently assessed the responses from each LLM to the furcation defect treatment questions. These blinded evaluations used a standardized rubric to score the answers (0–10) based on their comprehensiveness, scientific accuracy, clarity, and relevance [29] in relation to the established “gold standard.” Each LLM was queried once per furcation defect question on 13 December 2024, without any follow-up. The same evaluators re-scored the answers after a one-month interval to determine the consistency of their assessments.

### Statistical Analysis

The statistical analysis included descriptive statistics, Pearson and Spearman’s rho correlations, Cronbach’s α, and ICC to analyze the scores specifically for the furcation defect treatment questions and to assess inter-evaluator reliability. Wilcoxon’s and Friedman’s tests were employed to identify any statistically significant differences (*p* < 0.05) in the scores assigned by the different LLMs for these targeted questions. A Kruskal–Wallis test was performed to test the differences in the scores between the LLMs. All significance tests were evaluated at the 0.05 error level with a statistical software program (SPSS v.29.0, IBM, Armonk, NY, USA).

## 3. Results

Four LLMs—ChatGPT 4.0, Google Gemini, Google Gemini Advanced, and Microsoft Copilot—provided answers to ten clinical inquiries concerning the management and treatment of periodontal furcation defects, with their responses and the guideline/evidence-based “gold standard” detailed in Supplementary Table S1. The resulting answers were evaluated for comprehensiveness, scientific accuracy, clarity, and relevance, each scored from 0 to 10 by two independent specialists across two separate assessments conducted a month apart. Table 2 presents the descriptive statistics for these scores. Notably, Google Gemini Advanced received the highest average scores from both evaluators, while Google Gemini and Microsoft CoPilot received the lowest. 

Pearson and Spearman’s rho correlations, presented in Table 3, indicated strong and significant agreement between the two evaluators’ scores for each LLM across both scoring occasions, suggesting consistent evaluation [47,48]. Cronbach’s α and ICC tests further confirmed high inter-evaluator reliability (Table 4). Furthermore, statistical tests showed no significant differences in the scores the two evaluators gave, either in the initial or subsequent evaluation, or when the scores were combined (Table 5).

**Figure 1:**
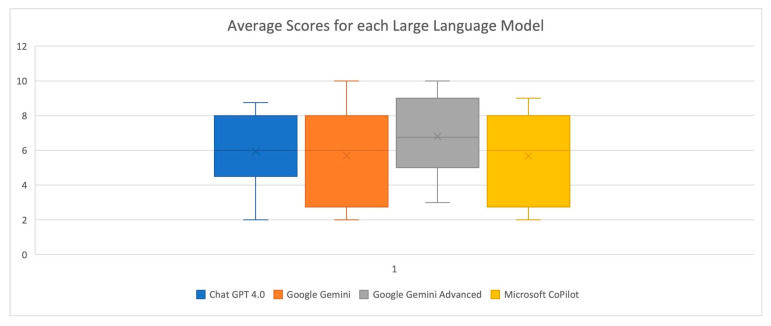
Average scores for each Large Language Model.

**Figure 2:**
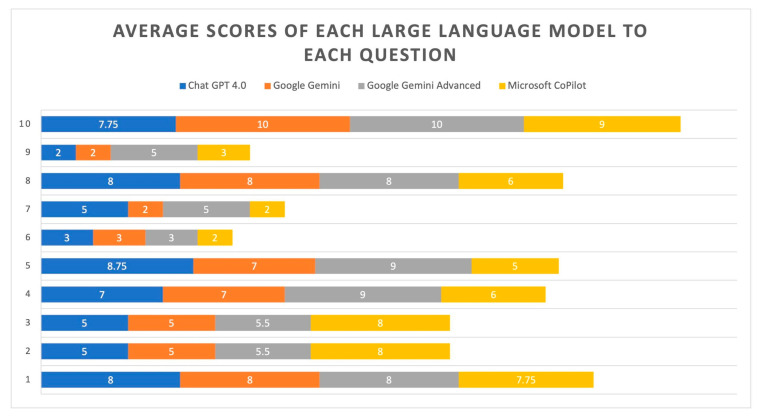
Average scores across the ten individual questions.

Consequently, an average score was calculated for each LLM’s response. As shown in Table 6, Google Gemini Advanced (6.80) achieved the highest average scores overall, while Google Gemini (5.70) and Microsoft CoPilot (5.68) exhibited the lowest scores. Figure 1 schematically illustrates the average scores for each LLM. Figure 2 demonstrates the average scores across the ten individual questions, with only responses to Questions 1 and 10 consistently scoring above seven for all LLMs. Based on the results presented in Table 7, the Kruskal–Wallis test showed no statistically significant differences in the average scores of answers provided by any of the LLM pairs (*p* > 0.05 for all comparisons). This indicates that for this specific analysis, the LLMs performed statistically similarly to each other in terms of their average scores.

Table 8 presents the descriptive statistics for the average scores assigned to answers from the four LLMs across four criteria: comprehensiveness, scientific accuracy, clarity, and relevance. The mean scores varied across LLMs and criteria, with Google Gemini Advanced generally achieving the highest mean scores for comprehensiveness and clarity. At the same time, Microsoft CoPilot tended to have slightly lower scores, particularly in relevance. The range of scores (minimum to maximum) indicates variability in the quality of answers within each LLM and across criteria. Overall, the LLMs demonstrated varying strengths and weaknesses across the assessed criteria, with no single LLM consistently outperforming the others in all categories; however, there is a trend for Google Gemini Advanced to score higher in comprehensiveness and clarity, and a trend for Microsoft CoPilot to score lower in relevance.

## 4. Discussion

The integration of AI in healthcare presents both opportunities and challenges. This study investigated the accuracy of four LLMs in answering common clinical inquiries concerning the management and treatment of periodontal furcation defects questions, comparing their responses to the “gold standard” of the EFP’s S3 Clinical Practice Guideline [38] and recent systematic reviews and meta-analyses [39,40,41,42,43,44,45,46]. The main findings of the present investigation were as follows:

- Google Gemini Advanced achieved the highest average scores, while Google Gemini and Microsoft Copilot received the lowest.
- The Kruskal–Wallis test showed no statistically significant differences in the average scores among the LLMs.
- Overall, the LLMs demonstrated varying strengths and weaknesses across the assessed criteria, with no single LLM consistently outperforming the others in all categories; however, there was a trend for Google Gemini Advanced to score higher in comprehensiveness and clarity, and a trend for Microsoft CoPilot to score lower in relevance.

In the present investigation, none of the pairwise comparisons between the LLMs showed a statistically significant difference in the average scores of their answers. All the adjusted *p*-values were very high (1.000), exceeding the typical significance level of 0.05. This indicates that based on this statistical test, the LLMs performed similarly regarding their average scores. Google Gemini Advanced tended to have the highest mean and median scores compared to the other LLMs, suggesting that on average, the evaluators rated its answers higher. Google Gemini and Microsoft CoPilot generally showed the lowest mean scores, indicating that their answers were rated somewhat lower on average. The standard deviations and variances indicate some variability in the scores for all the LLMs, showing that the quality of answers was not entirely uniform. Google Gemini exhibited the highest standard deviation (2.75) and variance (7.57), suggesting the greatest variability in its answers. Google Gemini Advanced displayed the lowest standard deviation (2.29) and variance (5.23), indicating the least variability. All the LLMs had a minimum score of 2.00, except for Google Gemini Advanced, which has a minimum of 3.00. The maximum scores ranged from 8.75 to 10.00.

The LLMs’ performance varied significantly across the ten questions. Some questions (Questions 1 and 10) generally received higher scores across all LLMs, while others (Questions 2, 6, and 7) tended to receive lower scores. This indicates that the quality of LLM-generated answers is affected by the inherent complexity or specific characteristics of the questions posed. Across most questions, Google Gemini Advanced demonstrated strong performance, often securing the highest or consistently high scores within each question group. This indicates a relatively consistent and strong performance. Microsoft CoPilot showed the most variability. While it performs reasonably well on some questions (Question 10), it tends to have lower scores on several others, suggesting less consistent performance. In addition, ChatGPT 4.0 showed a mixed performance, with some high scores and some moderate scores depending on the question. Google Gemini tended to have more moderate to lower scores than Google Gemini Advanced, indicating a difference in performance between the two Google LLMs. The varying scores across questions suggest that LLMs perform better on simpler inquiries (like Questions 1 and 10) and struggle with more complex or nuanced questions (such as Questions 2, 6, and 7).

Google Gemini Advanced demonstrated the strongest performance in comprehensiveness, suggesting that its answers were generally more thorough. Microsoft CoPilot tended to provide less comprehensive responses compared to the other LLMs. ChatGPT 4.0 and Google Gemini showed intermediate levels of comprehensiveness in their answers. Regarding scientific accuracy, Google Gemini Advanced exhibited relatively high scores, indicating a tendency to provide accurate information. Microsoft CoPilot’s responses showed the lowest average scores in this category, implying potential inaccuracies. The variability in standard deviations suggests inconsistencies in scientific accuracy across LLMs.

Furthermore, Google Gemini Advanced consistently performed well in terms of clarity, delivering answers that were generally well-articulated and easy to understand. While still reasonably clear, Microsoft CoPilot’s responses scored slightly lower on average than Google Gemini Advanced. Although some variability exists, all the LLMs showed a degree of consistency in clarity. Concerning relevance, Google Gemini Advanced maintained a relatively strong performance, indicating that its answers were generally pertinent to the questions. Microsoft CoPilot exhibited the lowest average scores for relevance, suggesting that its responses were sometimes less focused or off-topic. Google Gemini also showed the lowest mean in relevance.

A thorough examination of the existing literature indicated a scarcity of research evaluating and comparing the precision of LLMs in addressing typical clinical inquiries concerning the management and treatment of periodontal furcation defects, particularly when responses were measured against a “gold standard”. In a separate investigation, ChatGPT 4.0, Google Gemini, Google Gemini Advanced, and Microsoft Copilot were assessed for their effectiveness in responding to clinical questions pertinent to peri-implant disease [36]. This prior study involved presenting the LLMs with ten open-ended questions centered on the prevention and treatment of peri-implantitis and peri-implant mucositis. The outcomes showed that Google Gemini Advanced yielded the strongest performance, whereas Google Gemini exhibited the weakest. These findings align with the results observed in the current study. Furthermore, a distinct study focusing broadly on periodontology employed a comparable methodology, where clinical questions were posed to LLMs and periodontists then evaluated the responses based on comprehensiveness, scientific accuracy, clarity, and relevance; this research concluded that ChatGPT 4.0 generally demonstrated the best performance, while Google Gemini performed the least effectively [35].

Studies evaluating LLMs in dentistry show varied performance across specialties and models. In endodontics, ChatGPT demonstrated 57.3% accuracy and 85.4% consistency for dichotomous questions [31]. For oral and maxillofacial surgery, ChatGPT showed potential for patient-oriented responses (scoring 4.6 ± 0.8 on a 1–5 scale) but performed less effectively for technical answers (scoring 3.1 ± 1.5) [49]. When assessed on a board-style multiple-choice dental knowledge test, ChatGPT achieved a 76.9% correct answer rate, indicating competence in producing accurate dental content [50]. Similarly, ChatGPT 4.0 showed good accuracy for open-ended questions in head and neck surgery and provided accurate and current information regarding frequently asked questions about dental amalgam and its removal [51].

Comparisons between different LLMs within dentistry yield mixed results. One study found that ChatGPT-4 statistically outperformed ChatGPT-3.5, Bing Chat, and Bard [52]. In pediatric dentistry, ChatGPT achieved the highest accuracy among various LLMs, and the authors suggested that LLMs could be adjuncts in dental education and patient information distribution [53]. However, another study found no significant differences between ChatGPT and Google Bard when generating questions related to dental caries [9]. In oral and maxillofacial radiology examinations, ChatGPT, ChatGPT Plus, Bard, and Bing Chat generally performed unsatisfactorily, though ChatGPT Plus showed increased accuracy for basic knowledge [28]. Conversely, for clinically relevant orthodontic questions, Microsoft Bing Chat scored highest, outperforming ChatGPT 3.5 and Google Bard [29]. In endodontics, GPT-3.5 provided more credible information compared to Google Bard and Bing [37]. Overall, the diverse methodologies and dental specialties examined make direct comparisons of LLM performance challenging.

Several limitations within the study warrant consideration when interpreting its findings. Primarily, LLM performance can fluctuate based on question phrasing and the technical complexity of anticipated answers. This underscores the necessity for further research into how various question types impact LLMs’ capacity to produce accurate and pertinent information. To ensure a controlled comparison of LLM responses and mitigate potential biases stemming from uncontrolled elements like question quantity, wording, and specificity, follow-up questions were deliberately omitted. Although our methodology used a single input prompt per question to facilitate a controlled comparison and minimize biases from iterative prompting, it is recognized that this does not entirely mirror real-world user interactions, where follow-up questions are frequent and can affect the quality of LLM outputs. Open-ended questions can lead to inaccurate, irrelevant, or biased responses from LLMs because of the difficulty in evaluating and controlling the output. However, open-ended questions better mirror the type of inquiries clinicians typically use in practice, which are often not simple multiple-choice questions. They allow for a more natural interaction with the LLMs, similar to how a user might pose a question to a chatbot. In addition, the quality and scope of an LLM’s training data directly impacts its ability to analyze complex questions, potentially resulting in biased or incomplete conclusions. While we utilized a robust “gold standard” for evaluation, the inherent limitations of LLMs, such as their training data cutoffs and the unavailability of paywalled scientific information, are crucial considerations that may contribute to the observed variability in response quality and scientific accuracy.

The responses generated by the LLMs underwent independent evaluation on two separate occasions by two periodontists certified by the American Board of Periodontology. This assessment was conducted against a predefined criterion standard, and the process established high correlation and intra-evaluator reliability. This method minimizes the likelihood of individual bias influencing the scoring, given that the evaluations rely on the expert judgment of independent specialists. To comprehensively address various facets of furcation defect management and treatment, a set of 10 questions was chosen and presented to the LLMs. The study design incorporated a rigorous methodology, utilizing a predefined rubric to assess the LLM-generated answers for comprehensiveness, scientific accuracy, clarity, and relevance, enhancing the objectivity and reliability of the findings. This ensures that the assessment of the LLM-generated answers is structured and consistent. It reduces subjectivity, as the evaluators had clear benchmarks to follow instead of relying solely on their personal impressions. The open-ended questions allowed for a more in-depth evaluation of the LLM’s capabilities. It enabled us to assess not just the accuracy of a single fact, but also the comprehensiveness, clarity, and relevance of the information provided.

The clinical relevance of evaluating LLMs in dentistry is underscored by the challenging nature of managing periodontal furcation defects, a common issue where diverse treatment strategies exist and accurate information is crucial for optimal patient outcomes. This study provides a “snapshot” evaluation of current, widely accessible LLMs (ChatGPT 4.0, Google Gemini, Google Gemini Advanced, and Microsoft Copilot), acknowledging that while newer versions constantly emerge, this point-in-time assessment is vital for establishing benchmarks and understanding their evolving capabilities in healthcare. Despite the rapid advancements, this research offers forward-looking insights by identifying current strengths and weaknesses that can inform future LLM development, guide responsible clinical adoption, and highlight persistent challenges that necessitate ongoing human oversight in AI-assisted decision-making in dentistry.

To advance this field, subsequent investigations ought to explore diverse clinical scenarios, employing varied question formats. Further efforts could involve refining and validating these models to ensure more secure information accessibility and to facilitate the development of clinical recommendations, ultimately enhancing patient care. While Large Language Models possess significant potential to aid both patients and dental professionals, forthcoming studies should specifically investigate how this technology can contribute to better patient experiences and improved treatment outcomes.

## 5. Conclusions

The present investigation evaluated the performance of four LLMs in answering clinical questions related to the management and treatment of periodontal furcation defects. The findings indicate that while LLMs demonstrate potential in this area, their performance varies across different models and evaluation criteria. Google Gemini Advanced generally exhibited the strongest performance, particularly in comprehensiveness and clarity, whereas Google Gemini and Microsoft CoPilot tended to score lower. However, the overall statistical analysis revealed no significant differences in the average scores among the LLMs, suggesting a degree of similarity in their performance. Variability in LLM responses was observed across individual questions, highlighting the influence of question complexity and nature on answer quality.

## Figures and Tables

**Table 1:** Open-ended questions answered using ChatGPT 4.0, Google Gemini, Google Gemini Advanced, and Microsoft CoPilot.

Question Number	Question Description
1	How should molars with class II and III furcation involvement and residual pockets be best managed?
2	What is the best treatment option for residual deep pockets associated with mandibular Class II furcation involvement?
3	What is the best treatment option for residual deep pockets associated with maxillary buccal Class II furcation involvement?
4	What is the best choice of regenerative biomaterials for the regenerative treatment of residual deep pockets associated with Class II mandibular and maxillary buccal furcation involvement?
5	What is the best treatment option for maxillary interdental Class II furcation involvement?
6	What is the best treatment option for maxillary Class III furcation involvement?
7	What is the best treatment option for mandibular Class III furcation involvement?
8	Does adjunctive use of local drugs to subgingival instrumentation improve the clinical outcomes of furcation involvement?
9	Does adjunctive use of systemic antibiotics improve the clinical outcomes of furcation involvement?
10	What is the best imaging technique for assessing furcation defects?

**Table 2:** Descriptive statistics for scores given by 2 evaluators to answers provided by ChatGPT 4.0, Google Gemini, Google Gemini Advanced, and Microsoft CoPilot at two different times.

	Score 1								Score 2							
	ChatGPT 4.0		Google Gemini		Google Gemini Advanced		Microsoft CoPilot		ChatGPT 4.0		Google Gemini		Google Gemini Advanced		Microsoft CoPilot	
Evaluator	1	2	1	2	1	2	1	2	1	2	1	2	1	2	1	2
Mean	6.0	6.0	5.7	5.7	6.9	6.7	5.7	5.7	5.8	6.0	5.7	5.7	6.9	6.7	5.7	5.6
Standard Error of Mean	0.8	0.8	0.9	0.9	0.7	0.8	0.8	0.8	0.7	0.8	0.9	0.9	0.7	0.8	0.8	0.8
Median	6.0	6.0	6.0	6.0	7.0	6.5	6.0	6.0	6.0	6.0	6.0	6.0	7.0	6.5	6.0	6.0
Minimum	2.0	2.0	2.0	2.0	3.0	3.0	2.0	2.0	2.0	2.0	2.0	2.0	3.0	3.0	2.0	2.0
Maximum	9.0	9.0	10.0	10.0	10.0	10.0	9.0	9.0	8.0	9.0	10.0	10.0	10.0	10.0	9.0	9.0
Standard deviation	2.4	2.4	2.8	2.8	2.2	2.4	2.6	2.6	2.2	2.4	2.8	2.8	2.2	2.4	2.6	2.6
Variance	5.6	5.6	7.6	7.6	5.0	5.6	6.9	6.9	4.6	5.6	7.6	7.6	5.0	5.6	6.9	6.5

**Table 3:** Correlation between scores given by 2 evaluators to answers provided by ChatGPT 4.0, Google Gemini, Google Gemini Advanced, and Microsoft CoPilot at two different times. Pearson and Spearman’s rho correlations indicated strong and significant agreement between the two evaluators’ scores for each LLM across both scoring occasions, suggesting consistent evaluation.

Large Language Models (LLMs) [Evaluator 1–2]	Score 1		Score 2	
	Pearson Correlation	Spearman Rho	Pearson Correlation	Spearman Rho
ChatGPT 4.0	1.000 (*p* < 0.001)	1.000 (-)	0.987 (*p* < 0.001)	0.965 (*p* < 0.001)
Google Gemini	1.000 (*p* < 0.001)	1.000 (-)	1.000 (*p* < 0.001)	1.000 (-)
Google Gemini Advanced	0.985 (*p* < 0.001)	0.975 (*p* < 0.001))	0.985 (*p* < 0.001)	0.975 (*p* < 0.001)
Microsoft CoPilot	1.000 (*p* < 0.001)	1.000 (-)	0.993 (*p* < 0.001)	0.991 (*p* < 0.001)

**Table 4:** Cronbach α and Interclass Correlation Coefficient (ICC) for scores given by 2 evaluators to answers provided by ChatGPT 4.0, Google Gemini, Google Gemini Advanced, and Microsoft CoPilot at 2 different times and pooled scores of 2 different occasions. Cronbach’s α and ICC tests further confirmed high inter-evaluator reliability.

Large Language Models (LLMs)	Score 1			Score 2			Pooled Scores 1 and 2		
	Cronbach α	Interclass Correlation Coefficient		Cronbach α	Interclass Correlation Coefficient		Cronbach α	Interclass Correlation Coefficient	
		Single	Average		Single	Average		Single	Average
ChatGPT 4.0	1.000	1.000 (*p* < 0.001)	1.000 (*p* < 0.001)	0.991	0.983 (*p* < 0.001)	0.991 (*p* < 0.001)	1.000	1.000 (*p* < 0.001)	1.000 (*p* < 0.001)
Google Gemini	1.000	1.000 (*p* < 0.001)	1.000 (*p* < 0.001)	1.000	1.000 (*p* < 0.001)	1.000 (*p* < 0.001)	1.000	1.000 (*p* < 0.001)	1.000 (*p* < 0.001)
Google Gemini Advanced	0.992	0.983 (*p* < 0.001)	0.992 (*p* < 0.001)	0.992	0.983 (*p* < 0.001)	0.992 (*p* < 0.001)	1.000	1.000 (*p* < 0.001)	1.000 (*p* < 0.001)
Microsoft CoPilot	1.000	1.000 (*p* < 0.001)	1.000 (*p* < 0.001)	0.996	0.993 (*p* < 0.001)	0.996 (*p* < 0.001)	1.000	1.000 (*p* < 0.001)	1.000 (*p* < 0.001)

**Table 5:** Wilcoxon test for scores given by 2 evaluators to answers provided by ChatGPT 4.0, Google Gemini, Google Gemini Advanced, and Microsoft CoPilot at 2 different times. Friedman test for pooled scores given by 2 evaluators. Statistical analysis indicated no significant differences in scores given by the two evaluators across initial, subsequent, and combined evaluations.

Large Language Models (LLMs) [Evaluator 1–2]	Wilcoxon Test		Friedman Test Pooled Scores 1 and 2
	Score 1	Score 2	
ChatGPT 4.0	1.000	0.157	1.000
Google Gemini	1.000	1.000	1.000
Google Gemini Advanced	0.157	0.157	1.000
Microsoft CoPilot	1.000	0.317	1.000

**Table 6:** Descriptive statistics for average scores of answers provided by ChatGPT 4.0, Google Gemini, Google Gemini Advanced, and Microsoft CoPilot.

Average Score	ChatGPT 4.0	Google Gemini	Google Gemini Advanced	Microsoft CoPilot
Mean	5.95	5.70	6.80	5.68
Standard Error of Mean	0.73	0.87	0.72	0.82
Median	6.00	6.00	6.75	6.00
Minimum	2.00	2.00	3.00	2.00
Maximum	8.75	10.00	10.00	9.00
Standard deviation	2.30	2.75	2.29	2.60
Variance	5.29	7.57	5.23	6.78

**Table 7:** Kruskal–Wallis test for average scores of answers provided by ChatGPT 4.0, Google Gemini, Google Gemini Advanced, and Microsoft CoPilot.

Large Language Models (LLMs) [Average Scores]	Kruskal–Wallis (Adjusted *p*-Value by Bonferroni Correction for Multiple Tests)
ChatGPT 4.0 vs. Google Gemini	0.870 (1.000)
ChatGPT 4.0 vs. Google Gemini Advanced	0.329 (1.000)
ChatGPT 4.0 vs. Microsoft CoPilot	0.938 (1.000)
Google Gemini vs. Google Gemini Advanced	0.254 (1.000)
Google Gemini vs. Microsoft CoPilot	0.931 (1.000)
Google Gemini Advanced vs. Microsoft CoPilot	0.292 (1.000)

**Table 8:** Descriptive statistics for the average scores to the answers provided by ChatGPT 4.0, Google Gemini, Google Gemini Advances, and Microsoft CoPilot based on the examined criteria including comprehensiveness (1), scientific accuracy (2), clarity (3), and relevance (4).

	ChatGPT 4.0				Google Gemini				Google Gemini Advanced				Microsoft CoPilot			
Criteria examined	1	2	3	4	1	2	3	4	1	2	3	4	1	2	3	4
Mean	6.0	6.2	6.0	5.2	5.7	5.8	5.9	5.0	7.3	7.0	7.0	6.8	5.6	5.4	5.8	5.9
Standard Error of Mean	0.7	0.8	0.7	0.9	0.9	0.9	0.9	1.1	0.6	0.7	0.7	1.0	0.8	0.9	0.8	1.0
Median	5.5	6.0	6.0	6.0	6.0	6.0	6.5	4.5	7.8	7.0	7.0	6.8	6.0	6.0	6.0	6.8
Minimum	2.0	1.5	2.0	2.0	2.0	2.0	2.0	1.5	4.0	4.0	3.0	2.0	2.0	1.5	2.0	1.0
Maximum	9.0	9.0	9.0	8.5	10.0	10.0	10.0	10.0	10.0	10.0	10.0	10.0	9.0	9.0	9.0	10.0
Standard deviation	2.2	2.5	2.3	2.9	2.8	2.7	2.7	3.3	2.0	2.1	2.3	3.1	2.6	2.9	2.6	3.0
Variance	4.9	6.0	5.4	8.2	7.6	7.3	7.4	11.0	4.0	4.2	5.3	9.8	6.5	8.7	6.9	9.0

## Data Availability

Data are available upon reasonable request.

## Supplementary Materials

The following supporting information can be downloaded at https://www.mdpi.com/article/10.3390/dj13060271/s1, Supplemental Table S1: The ten indicative questions relevant to common clinical issues concerning the management and treatment of periodontal furcation defects were asked to four different Large Language Models.

## Abbreviations

The following abbreviations are used in this manuscript:

AI	Artificial Intelligence
LLM	Large Language Model
