# Supplementary material for: Evaluation of Large Language Model Performance in Answering Clinical Questions on Periodontal Furcation Defect Management
Source: Dent J (Basel). 2025 Jun 18;13(6):271. doi: 10.3390/dj13060271 (PMC12191798; doi:10.3390/dj13060271)
Supplement: Supplementary file 1 [file dentistry-13-00271-s001.zip › dentistry-3693744-supplementary.pdf]

Supplemental Table 1: The ten indicative questions relevant to common clinical issues concerning the management and treatment of periodontal furcation defects asked to four different large language models

QUESTION	Chat GPT 4.0	GEMINI	GEMINI ADVANCED	MICROSOFT COPILOT	EVIDENCE
**How should molars with class II and III furcation involvement and residual pockets be best managed?**	Managing molars with Class II and III furcation involvement and residual pockets requires a comprehensive approach that may include the following steps:<br><br>1. Assessment<br><br>Clinical Evaluation: Assess the extent of furcation involvement and periodontal pockets.<br><br>Radiographic Evaluation: Use radiographs to evaluate bone loss and furcation involvement.<br><br>2. Non-Surgical Therapy<br><br>Scaling and Root Planing: Thoroughly clean the root surfaces to reduce bacterial load.<br><br>Antimicrobial Therapy: Consider local delivery of antimicrobials or systemic antibiotics if indicated.<br><br>3. Surgical Management<br><br>Flap Surgery: If pockets remain after non-surgical therapy, flap surgery may be performed to access and debride the furcation areas.<br><br>Regenerative Procedures: Use of bone grafts, membranes, or growth factors to	Molars with Class II and III furcation involvement and residual pockets present a significant challenge in periodontics. The management of these cases requires careful assessment and a tailored treatment plan. Here are some key considerations:<br><br>1. Assessment and Prognosis:<br><br>Severity of furcation involvement: Class II and III defects indicate a more advanced stage of periodontal disease.<br><br>Residual pockets: These can harbor bacteria and contribute to further tissue destruction.<br><br>Tooth and bone support: The overall health of the tooth and surrounding bone structure is crucial.<br><br>2. Treatment Options:<br><br>a. Non-Surgical Therapy:<br><br>Scaling and root planing: Thorough cleaning to remove plaque and tartar.<br><br>Antibiotics: May be prescribed to address bacterial infections.	Management of molars with class II and III furcation involvement and residual pockets is complex and depends on several factors, including:<br><br>Specifics of the furcation involvement: Depth, configuration, and accessibility of the furcation defect.<br><br>Residual pocket depth and location: Severity of inflammation and location of the remaining pockets.<br><br>Tooth's strategic importance: Is it crucial for maintaining function and occlusion?<br><br>Patient's overall oral health: Including oral hygiene, systemic health, and compliance.<br><br>Restorative considerations: Can the tooth be restored adequately?<br><br>Here's a breakdown of the general approach:<br><br>1. Non-Surgical Therapy	Managing molars with Class II and III furcation involvement and residual pockets requires a comprehensive approach. Here are some key steps:<br><br>Non-Surgical Treatment: This includes scaling and root planing to remove plaque and calculus from the root surfaces. This helps reduce inflammation and deepens the pockets<br><br>Home Care: Patients should be educated on proper oral hygiene techniques to maintain the health of their teeth and gums. This includes regular brushing, flossing, and possibly using interdental brushes or water flossers.<br><br>Regular Maintenance: Periodic professional cleanings and check-ups are essential to monitor the condition and prevent further progression of the disease.<br><br>Surgical Intervention: In cases where non-surgical treatments are not sufficient, surgical options such as flap surgery, bone grafts, or	We recommend that molars with Class II and III furcation involvement and residual pockets receive periodontal therapy.<br><br>Furcation involvement is no reason for extraction.

	promote bone regeneration in Class II and III furcations.<br><br>Tunnel Procedures: For Class II furcations, tunneling may improve access and maintenance.<br><br>4. Maintenance<br><br>Regular Recall: Schedule frequent follow-up visits to monitor periodontal health and perform maintenance cleanings.<br><br>Patient Education: Teach patients proper oral hygiene techniques tailored to their condition, including the use of interdental brushes or other aids for furcation areas.<br><br>5. Prosthetic Considerations<br><br>In some cases, extraction may be considered if the tooth is non-restorable or if periodontal treatment is unsuccessful. Replacement options should be discussed with the patient, such as implants or bridges.<br><br>6. Multidisciplinary Approach<br><br>Collaborate with periodontists, oral surgeons, and restorative dentists to create a comprehensive treatment plan tailored to the individual patient's needs.<br><br>Each case should be evaluated individually to determine the best management strategy based on the severity of the furcation involvement, patient compliance, and overall periodontal health.	Periodontal maintenance: Regular follow-up visits for cleaning and evaluation.<br><br>b. Surgical Therapy:<br><br>Open flap debridement: Surgical access to the furcation area for deep cleaning and root smoothing.<br><br>Regenerative procedures: Bone grafting and guided tissue regeneration (GTR) may be considered to fill the defect and promote tissue growth.<br><br>Root amputation: In severe cases, a portion of the root may be removed to improve accessibility and reduce the risk of fracture.<br><br>Extraction: If the tooth is non-restorable or the prognosis is poor, extraction may be necessary.<br><br>3. Factors to Consider:<br><br>Patient's overall health: Systemic conditions can influence treatment options.<br><br>Tooth's function: The importance of the tooth in the patient's bite and chewing function should be assessed.<br><br>Cost and complexity: The patient's financial situation and the complexity of the procedure should be considered.<br><br>4. Long-Term Management:<br><br>Supportive periodontal therapy (SPT): Regular checkups and cleanings are essential to maintain the health of the treated tooth.<br><br>Risk factors: Patients should be advised to address any risk factors, such as smoking or poor oral hygiene.	Scaling and root planing: Thorough cleaning to remove plaque and calculus from the root surfaces and within the furcation defect.<br><br>Oral hygiene instructions: Reinforce proper brushing and interdental cleaning techniques, possibly recommending specialized tools like interdental brushes.<br><br>Antimicrobial therapy: May include local delivery of antimicrobials (e.g., chlorhexidine chips) or systemic antibiotics in cases of severe infection.<br><br>2. Surgical Therapy (if non-surgical therapy is insufficient)<br><br>Open flap debridement: Allows for direct visualization and cleaning of the furcation area, and may include osseous recontouring to eliminate the defect.<br><br>Guided tissue regeneration (GTR): Uses a barrier membrane to encourage regeneration of periodontal tissues in the furcation.<br><br>Bone grafting: May be used to fill the furcation defect and promote bone regeneration.<br><br>Furcation plasty: Reshaping the tooth structure to improve access for cleaning.<br><br>Root resection/hemisection: Removing one or more roots of the molar to eliminate the furcation involvement. This is more common with Class III involvement.<br><br>3. Extraction	guided tissue regeneration may be considered. These procedures aim to reduce pocket depth and regenerate lost bone and tissue.<br><br>Specialist Referral: For complex cases, referral to a periodontist (a specialist in gum disease) may be necessary for advanced treatment options and management.<br><br>Patient Education: Informing the patient about the condition, treatment options, and the importance of adherence to the treatment plan is crucial for successful outcomes<br><br>It's important to tailor the treatment plan to the individual patient's needs and preferences. Regular follow-ups and adjustments to the plan may be needed based on the patient's response to treatment.	

		Note: The optimal treatment plan for each case will depend on individual factors. A consultation with a periodontist is recommended to discuss the specific options and potential outcomes.	In some cases, especially with advanced Class III furcation involvement, severe bone loss, or if the tooth is not restorable, extraction may be the best option.<br><br>4. Maintenance<br><br>Regular periodontal maintenance: Essential to monitor the treated tooth and prevent recurrence of disease. This includes professional cleanings and ongoing home care.<br><br>Important Notes:<br><br>Prognosis: Teeth with furcation involvement have a guarded prognosis, even with treatment.<br><br>Patient education: Clear communication with the patient about the condition, treatment options, and expected outcomes is crucial.<br><br>It's critical to consult with a periodontist (a dentist specializing in gum diseases and their treatment) to determine the most appropriate treatment plan for your specific situation. They can perform a thorough examination, assess the factors mentioned above, and recommend the best course of action to preserve your oral health.		
**What is the best treatment option for residual deep pockets associated with mandibular Class II**	For residual deep pockets associated with mandibular Class II furcation involvement, the best treatment option often includes a combination of non-surgical and surgical approaches:<br><br>1. Non-Surgical Therapy	Mandibular Class II furcation involvement presents a unique challenge due to the anatomy of the mandible. The deep pockets in this area can be difficult to access and treat. Here are some potential treatment options:	The best treatment option for residual deep pockets associated with mandibular Class II furcation involvement depends on several factors, including the specific characteristics of the furcation involvement, the overall periodontal health of the patient, and their individual preferences. However, here's a breakdown of the general	For residual deep pockets associated with mandibular Class II furcation involvement, periodontal regenerative surgery is often recommended.This approach aims to reduce pocket depth and regenerate lost bone and tissue	We recommend treating mandibular molars with residual pockets associated with Class II furcation involvement with periodontal regenerative surgery.

**furcation involvement?**	Scaling and Root Planing (SRP): Initial therapy should involve thorough scaling and root planing to remove biofilm and calculus from the root surfaces.<br><br>Antimicrobial Therapy: Consider the use of local antimicrobials or systemic antibiotics if there’s evidence of significant infection.<br><br>2. Surgical Therapy<br><br>Flap Surgery: If pockets remain deep after non-surgical treatment, flap surgery may be indicated. This allows direct access to the furcation area for debridement and access for improved maintenance.<br><br>Gingival Recession Procedures: If needed, procedures to enhance the attached gingiva may also be beneficial.<br><br>Bone Regeneration: For Class II furcations, consider regenerative techniques such as bone grafts or guided tissue regeneration (GTR) to promote bone fill in the furcation area.<br><br>3. Maintenance<br><br>Regular Monitoring: Frequent recall visits to monitor periodontal health and provide maintenance cleanings are crucial.<br><br>Patient Education: Emphasize the importance of home care, including the use of interdental brushes or soft picks designed for furcation access.<br><br>4. Consider Extraction<br><br>If the tooth has a poor prognosis despite treatment efforts, extraction may be necessary, followed by discussion of potential prosthetic replacements.<br><br>5. Multidisciplinary Approach	1. Surgical Therapy:<br><br>Open Flap Debridement: This involves surgically accessing the furcation area to remove plaque and tartar and smooth the root surfaces.<br><br>Gingival Grafting: This procedure can help to create a thicker gingival attachment around the tooth, which can improve the health of the surrounding tissues.<br><br>Guided Tissue Regeneration (GTR): This technique uses a barrier membrane to guide the growth of new bone and connective tissue into the defect.<br><br>Root Amputation: In severe cases, a portion of the root may be removed to improve access to the furcation area.<br><br>2. Non-Surgical Therapy:<br><br>Scaling and Root Planing: This is a deep cleaning procedure that can help to remove plaque and tartar from the root surfaces.<br><br>Antibiotics: In some cases, antibiotics may be prescribed to help control infection.<br><br>Periodontal Maintenance: Regular checkups and cleanings are essential to maintain the health of the treated area.<br><br>Factors to Consider:<br><br>Severity of the furcation involvement: The extent of the damage will influence the choice of treatment.<br><br>Tooth and bone support: The overall health of the tooth and surrounding bone is important.<br><br>Patient's overall health: Systemic conditions may affect treatment options.	approach and some key considerations:<br><br>1. Initial Therapy:<br><br>Non-surgical periodontal therapy: This is always the first step and involves scaling and root planing to remove plaque and calculus from the root surfaces, both within the pocket and within the furcation area. This may be accompanied by adjunctive therapies like local antiseptics or antibiotics.<br><br>Re-evaluation: After the initial therapy, the pockets and furcation involvement are reassessed. If the deep pockets persist, further treatment is necessary.<br><br>2. Surgical Therapy:<br><br>Open flap debridement: This is often the preferred surgical approach for Class II furcation involvement. It involves creating a flap to access the roots and furcation area, allowing for thorough debridement and root surface modification. The flap is then repositioned to reduce pocket depth and improve access for oral hygiene.<br><br>Bone grafting: In some cases, bone grafting may be used to try to regenerate lost bone in the furcation area. This can help to improve the long-term prognosis of the tooth.<br><br>Guided tissue regeneration (GTR): GTR can be used in combination with bone grafting to further enhance bone regeneration. It involves placing a barrier membrane to prevent soft tissue from growing into the bone	Here are some common regenerative surgical options:<br><br>Flap Surgery: This involves lifting the gum tissue to access the roots and bone for thorough cleaning and reshaping<br><br>Bone Grafts: Bone grafts can be used to fill in areas where bone has been lost, promoting new bone growth.<br><br>Guided Tissue Regeneration (GTR): This technique uses barrier membranes to encourage the growth of new bone and gum tissue while preventing the growth of unwanted tissues.<br><br>It's important to consult with a periodontist to determine the most appropriate treatment plan based on the specific condition of the teeth and gums. Regular follow-ups and maintenance are crucial for the success of the treatment.	

	Collaborate with periodontists and restorative dentists to ensure a comprehensive treatment plan tailored to the patient’s specific needs.<br><br>Ultimately, the choice of treatment should be individualized based on the patient’s overall periodontal status, compliance, and treatment response.	Cost and complexity: The patient's financial situation and the complexity of the procedure should be considered.<br><br>It's important to consult with a periodontist to discuss the best treatment options for your specific case. They will be able to assess the severity of the furcation involvement and recommend the most appropriate approach.	defect, allowing bone cells to repopulate the area.<br><br>Furcation plasty: This procedure involves reshaping the tooth structure in the furcation area to make it more accessible for cleaning. It can be used in conjunction with open flap debridement.<br><br>Tunnel preparation: This technique creates a "tunnel" through the furcation area, allowing for better access for cleaning. It may be considered in specific cases.<br><br>Root resection/hemisection: In some cases where the furcation involvement is severe or the tooth is otherwise compromised, root resection or hemisection may be considered. This involves removing one or more roots of the tooth, allowing the remaining portion to be retained.<br><br>Extraction: As a last resort, if the tooth cannot be saved with other treatments, extraction may be necessary.<br><br>Key Considerations:<br><br>Patient factors: The patient's overall health, oral hygiene habits, and compliance with treatment will influence the choice of therapy and the long-term prognosis.<br><br>Anatomical factors: The specific morphology of the furcation involvement, root anatomy, and surrounding bone levels will affect the treatment options.<br><br>Restorative considerations: The presence of existing restorations or the need for future restorative work will		

			also factor into the decision-making process.<br><br>Important Note:<br><br>Regular maintenance: Regardless of the chosen treatment, ongoing periodontal maintenance is crucial to prevent recurrence of the disease and maintain the long-term health of the teeth.<br><br>It's important to consult with a qualified periodontist to determine the best treatment option for your specific situation. They can perform a thorough examination, assess the severity of the furcation involvement, and discuss the risks and benefits of each treatment option.		
**What is the best treatment option for residual deep pockets associated with maxillary buccal Class II furcation involvement?**	For residual deep pockets associated with maxillary buccal Class II furcation involvement, the best treatment options typically include a combination of non-surgical and surgical approaches tailored to the specific condition of the tooth and the patient's overall periodontal health. Here's a detailed outline:<br><br>1. Non-Surgical Therapy<br><br>Scaling and Root Planing (SRP): Conduct thorough SRP to remove plaque and calculus from the root surfaces, including the furcation area.<br><br>Antimicrobial Therapy: Consider local delivery of antimicrobials (like chlorhexidine) or systemic antibiotics if there's significant inflammation or infection.<br><br>2. Surgical Therapy<br><br>Flap Surgery: If pockets remain deep after non-surgical treatment, flap	Treatment Options for Maxillary Buccal Class II Furcation Involvement<br><br>Maxillary buccal Class II furcation involvement is a challenging condition in periodontology, characterized by a partial exposure of the furcation anatomy on the buccal side of a maxillary molar. The best treatment option depends on various factors, including the severity of the involvement, the patient's overall oral health, and personal preferences.<br><br>Here are some common treatment options:<br><br>1. Surgical Periodontal Therapy<br><br>Open Flap Debridement: This involves surgically accessing the furcation area to remove plaque and calculus, and to smooth the root surface. It's often	Treating residual deep pockets with maxillary buccal Class II furcation involvement presents unique challenges due to the anatomy of the area. Here's a breakdown of the treatment considerations:<br><br>1. Initial Non-Surgical Therapy:<br><br>Scaling and root planing: Thoroughly remove plaque and calculus from the affected root surfaces, including within the furcation.<br><br>Adjunctive therapies: Consider local antiseptics (like chlorhexidine) or systemic antibiotics to improve initial healing.<br><br>2. Re-evaluation:<br><br>Assess pocket depths and furcation involvement: If deep pockets persist, surgical intervention is likely needed.<br><br>3. Surgical Therapy Options:	For residual deep pockets associated with maxillary buccal Class II furcation involvement, periodontal regenerative surgery is often the best treatment option<br><br>This approach aims to reduce pocket depth and regenerate lost bone and tissue<br><br>Here are some common regenerative surgical options:<br><br>Flap Surgery: This involves lifting the gum tissue to access the roots and bone for thorough cleaning and reshaping<br><br>Bone Grafts: Bone grafts can be used to fill in areas where bone has been lost, promoting new bone growth<br><br>Guided Tissue Regeneration (GTR): This technique uses	We suggest treating molars with residual pockets associated with maxillary buccal Class II furcation involvement with periodontal regenerative surgery.

	surgery can provide direct access for debridement of the furcation area.<br><br>Bone Regeneration Techniques: Consider using guided tissue regeneration (GTR) or bone grafting materials to promote regeneration of bone in the furcation area, especially if there is significant bone loss.<br><br>Root Resection: In some cases, if the furcation involvement is severe and the tooth is restorable, root resection might be an option. This involves removing the affected root while preserving the rest of the tooth.<br><br>3. Maintenance<br><br>Regular Follow-Up: Schedule frequent maintenance visits to monitor periodontal health and perform cleaning, especially focusing on the furcation areas.<br><br>Patient Education: Teach the patient effective home care techniques, including the use of interdental brushes or other aids to improve access to the furcation.<br><br>4. Consider Extraction<br><br>If the tooth prognosis remains poor despite treatment, extraction may be necessary. Discuss potential prosthetic options with the patient, such as implants or bridges.<br><br>5. Multidisciplinary Collaboration<br><br>Work closely with periodontists and restorative dentists to develop a comprehensive treatment plan that addresses all aspects of the patient's oral health.<br><br>By customizing the treatment approach based on the individual case, you can enhance the chances of successful management of the residual pockets and	combined with a bone graft or enamel matrix derivative (EMD) to promote tissue regeneration.<br><br>Guided Tissue Regeneration (GTR): This technique uses a barrier membrane to separate the gingival tissue from the bone, allowing for the growth of new periodontal tissue.<br><br>Furcation Flap Procedure: This involves creating a flap to access the furcation and either resecting the involved root or performing a root amputation.<br><br>2. Endodontic Therapy<br><br>If the tooth has a pulp infection or necrosis, root canal therapy may be necessary before or after periodontal surgery.<br><br>3. Extraction<br><br>In severe cases, extraction of the tooth may be recommended if the prognosis for long-term survival is poor.<br><br>Factors influencing the choice of treatment:<br><br>Severity of furcation involvement: Class II involvement may be treatable with surgical therapy, while Class III involvement often requires extraction.<br><br>Patient's overall oral health: A patient with good oral hygiene and systemic health may be a better candidate for surgical treatment.	Open flap debridement: This remains a primary approach. It allows for visualization and access to the furcation, facilitating thorough cleaning, root planing, and removal of diseased tissue.<br><br>Bone grafting: May be considered to attempt regeneration of lost bone, especially if the defect is contained. However, maxillary buccal furcations often have poorer blood supply, making grafting less predictable.<br><br>Guided tissue regeneration (GTR): Can be used with or without bone grafting to enhance regeneration by excluding epithelial tissue and encouraging bone and periodontal ligament cell growth.<br><br>Root resection: Resection of the buccal root may be an option if the palatal root is strong and can support the tooth. This simplifies the anatomy and improves cleansibility.<br><br>Challenges and Considerations:<br><br>Access and visibility: Maxillary molars can be difficult to access, especially the second and third molars.<br><br>Root anatomy: Maxillary molars have a complex root structure, with the buccal roots often being close together, making instrumentation and regeneration challenging.<br><br>Esthetics: Gingival recession following surgery is a concern in the maxillary anterior region, but less so for the molars. However, careful flap design and management are still crucial.<br><br>Factors Influencing Treatment Choice:<br><br>Severity of furcation involvement: The extent of bone loss and the	barrier membranes to encourage the growth of new bone and gum tissue while preventing the growth of unwanted tissues<br><br>Consulting with a periodontist is essential to determine the most appropriate treatment plan based on the specific condition of the teeth and gums<br><br>Regular follow-ups and maintenance are crucial for the success of the treatment.	

	improve the long-term prognosis for the affected tooth.	Tooth vitality: A non-vital tooth may require endodontic therapy before periodontal treatment.<br><br>Patient preferences: The patient's desire to retain the tooth and their willingness to undergo complex treatments should be considered.<br><br>It's important to consult with a periodontist to discuss the specific treatment options for your individual case. They will be able to assess the severity of the furcation involvement, evaluate the risks and benefits of different treatments, and recommend the best course of action based on your unique circumstances.	configuration of the furcation entrance will guide the decision.<br><br>Overall periodontal health: The patient's overall periodontal status, including plaque control and systemic factors, will influence the prognosis.<br><br>Restorative considerations: Existing restorations or the need for future restorative work may impact treatment options.<br><br>Patient preferences: The patient's understanding of the treatment options, their expectations, and their willingness to commit to long-term maintenance are important factors.<br><br>Important Note: Maintenance: Regular periodontal maintenance is critical after any treatment to prevent disease recurrence and maintain long-term oral health.		
**What is the best choice of regenerative biomaterials for the regenerative treatment of residual deep pockets associated with Class II mandibular and maxillary buccal furcation involvement?**	When considering regenerative treatment for residual deep pockets associated with Class II mandibular and maxillary buccal furcation involvement, several biomaterials are effective for promoting tissue and bone regeneration. The best choices include:<br><br>1. Bone Grafts<br><br>Autografts: Bone harvested from the patient's own body. This is the gold standard due to its osteogenic potential but may require additional surgical sites.<br><br>Allografts: Processed human bone from a donor, providing a scaffold for new bone formation.<br><br>Xenografts: Bone from non-human sources (like bovine) that are	The best choice of regenerative biomaterials for the regenerative treatment of residual deep pockets associated with Class II mandibular and maxillary buccal furcation involvement is a complex issue with no definitive answer. The optimal choice may vary depending on several factors, including:<br><br>The specific characteristics of the furcation defect: The size, shape, and location of the defect can influence the choice of biomaterial.<br><br>The patient's overall health and medical history: Certain medical conditions or medications may contraindicate the use of certain biomaterials.<br><br>The dentist's experience and preference: Different dentists may have different preferences for certain biomaterials.	The choice of regenerative biomaterial for deep pockets associated with Class II furcation involvement depends on several factors, including the specific defect morphology, patient factors, and clinician preference. However, some commonly used and effective options include:<br><br>1. Bone Grafts:<br><br>Autografts: Gold standard, taken from the patient's own body (e.g., iliac crest). Excellent osteoconductive and osteoinductive potential but limited availability and donor site morbidity.<br><br>Allografts: Derived from human cadavers, processed to minimize immune rejection. Good osteoconductivity, readily available,	The choice of regenerative biomaterials for treating residual deep pockets associated with Class II mandibular and maxillary buccal furcation involvement depends on the specific needs of the case and the preferences of the periodontist. Here are some commonly used regenerative biomaterials:<br><br>Demineralized Freeze-Dried Bone Allograft (DFDBA): This is a commonly used bone graft material that provides a scaffold for new bone growth1<br><br>.	We recommend treating molars with residual pockets associated with mandibular and maxillary buccal Class II furcation involvement with periodontal regenerative therapy using enamel matrix derivative alone or bone-derived graft with or without resorbable membranes*

	biocompatible and can provide a scaffold for regeneration.<br><br>Alloplasts: Synthetic materials (like calcium phosphates or hydroxyapatite) that can provide structural support and encourage bone growth.<br><br>2. Guided Tissue Regeneration (GTR) Membranes<br><br>Resorbable Membranes: Made from materials like polylactic acid (PLA) or polycaprolactone (PCL), these membranes support cell proliferation and tissue healing while being absorbed by the body over time.<br><br>Non-Resorbable Membranes: Typically made from materials like expanded polytetrafluoroethylene (ePTFE), these membranes can provide a physical barrier for a longer period, but require a second surgery for removal.<br><br>3. Growth Factors and Cytokines<br><br>Platelet-Rich Plasma (PRP): Concentrated platelets from the patient's blood, which release growth factors that promote healing and regeneration.<br><br>Bone Morphogenetic Proteins (BMPs): These proteins can enhance osteogenesis and are sometimes used in conjunction with graft materials.<br><br>4. Combination Products<br><br>Some products combine bone graft materials with growth factors (like allograft materials infused with BMPs) to enhance regenerative potential.<br><br>Considerations<br><br>Case Specificity: The choice of biomaterial may depend on factors such	That said, some of the most commonly used regenerative biomaterials for furcation defects include:<br><br>Enamel matrix proteins (EMPs): EMPs are derived from the enamel of developing teeth and have been shown to promote bone and tissue regeneration.<br><br>Bone grafts and substitutes: Bone grafts and substitutes can be used to fill in bone defects and promote new bone growth.<br><br>Barrier membranes: Barrier membranes can be used to create a space between the soft tissue and bone, allowing for the growth of new bone and tissue.<br><br>Growth factors: Growth factors can be used to stimulate the growth and differentiation of cells.<br><br>It is important to consult with a qualified periodontist to determine the best choice of regenerative biomaterial for your specific situation. The periodontist will be able to assess the characteristics of your furcation defect and recommend the most appropriate treatment.	but potential for disease transmission.<br><br>Xenografts: Derived from animal sources (e.g., bovine bone). Osteoconductive, readily available, but concerns about immunogenicity and disease transmission.<br><br>Alloplasts: Synthetic materials (e.g., calcium phosphate, bioactive glass). Osteoconductive, biocompatible, and available in various forms, but may lack osteoinductive properties.<br><br>2. Guided Tissue Regeneration (GTR) Membranes:<br><br>Resorbable: (e.g., collagen, synthetic polymers) Convenient, eliminate the need for a second surgery, but may have variable resorption rates and mechanical properties.<br><br>Non-resorbable: (e.g., expanded polytetrafluoroethylene - ePTFE) Provide good mechanical support and tissue separation, but require a second surgery for removal.<br><br>3. Growth Factors:<br><br>Platelet-derived growth factor (PDGF): Stimulates cell proliferation and migration, promoting wound healing and tissue regeneration.<br><br>Bone morphogenetic proteins (BMPs): Induce bone formation, enhancing bone regeneration in periodontal defects.<br><br>Enamel matrix derivative (EMD): Derived from developing tooth enamel, promotes periodontal regeneration, including cementum formation and periodontal ligament attachment.<br><br>4. Combination Products:	Enamel Matrix Derivative (EMD): This biomaterial promotes the formation of new cementum, periodontal ligament, and alveolar bone.<br><br>Platelet-Rich Plasma (PRP): PRP is derived from the patient's own blood and contains growth factors that promote healing and tissue regeneration.<br><br>Collagen Membranes: These are used in guided tissue regeneration (GTR) to promote the growth of new bone and prevent the ingrowth of unwanted tissues.<br><br>Bioactive Glass: This material has been shown to promote bone regeneration and has antimicrobial properties.<br><br>Each of these biomaterials has its own advantages and may be used alone or in combination, depending on the specific clinical situation. Consulting with a periodontist is essential to determine the most appropriate biomaterials for your case.	

	as the severity of bone loss, patient health, and surgical access.<br><br>Long-Term Outcomes: Research shows that a combination of GTR with bone graft materials often yields better results in furcation areas compared to either treatment alone.<br><br>In conclusion, a tailored approach using a combination of bone grafts, GTR membranes, and growth factors can enhance the success of regenerative treatments for Class II furcation involvement. Always consider the individual patient's condition and preferences when selecting materials.		Many commercially available products combine different biomaterials (e.g., bone graft with GTR membrane, growth factors with a carrier matrix) to optimize regenerative outcomes.<br><br>Specific Considerations for Class II Furcation Involvement:<br><br>Accessibility: Buccal furcations can be challenging to access, especially in the maxillary molars. Injectable or moldable biomaterials may be preferred in these cases.<br><br>Defect morphology: The depth and width of the furcation defect will influence the choice of biomaterial. Deeper and wider defects may benefit from a combination of bone graft and GTR membrane.<br><br>Patient factors: Systemic health, smoking status, and oral hygiene compliance should be considered when selecting a biomaterial.<br><br>Emerging Biomaterials:<br><br>Stem cell-based therapies: Mesenchymal stem cells (MSCs) show promise for periodontal regeneration, but further research is needed to optimize their clinical application.<br><br>3D-printed scaffolds: Can be customized to the specific defect morphology, providing a framework for tissue regeneration.<br><br>Conclusion:<br><br>The ideal biomaterial for regenerative treatment of Class II furcation involvement depends on a careful assessment of the individual case. A combination of biomaterials, such as a bone graft with a GTR membrane and/or growth factors, may be		

			necessary to achieve optimal results. Ongoing research and development of new biomaterials and techniques are continuously improving the possibilities for periodontal regeneration.<br><br>It is crucial to consult with a qualified periodontist to determine the most appropriate treatment plan and biomaterial selection for your specific needs.		
**What is the best treatment option for maxillary interdental Class II furcation involvement?**	The best treatment option for maxillary interdental Class II furcation involvement typically involves a combination of non-surgical and surgical approaches, tailored to the specific clinical situation. Here's a comprehensive treatment strategy:<br><br>1. Non-Surgical Therapy<br><br>Scaling and Root Planing (SRP): Perform thorough SRP to eliminate plaque and calculus from the root surfaces and the furcation area.<br><br>Antimicrobial Therapy: Consider the use of local antimicrobials (e.g., chlorhexidine) or systemic antibiotics if there is significant inflammation or infection.<br><br>2. Surgical Therapy<br><br>Flap Surgery: If deep pockets persist after non-surgical treatment, flap surgery may be indicated. This procedure allows for better access to the furcation area for debridement and improves visibility.<br><br>Guided Tissue Regeneration (GTR): Utilizing GTR techniques with resorbable or non-resorbable membranes can help promote regeneration of periodontal tissues and bone in the furcation area.	The best treatment option for maxillary interdental Class II furcation involvement depends on several factors, including:<br><br>The severity of the furcation involvement: Class II furcation involvement is considered moderate, but the specific extent of the bone loss can vary.<br><br>The patient's overall oral health: The presence of other periodontal diseases or conditions can influence the treatment plan.<br><br>The patient's preferences: The patient's desires and expectations for the outcome of treatment should also be considered.<br><br>Here are some potential treatment options for maxillary interdental Class II furcation involvement:<br><br>1. Surgical Periodontal Therapy:<br><br>Flap Procedures: These involve raising a flap of gum tissue to access the affected furcation. The bone and soft tissue can then be cleaned and reshaped	The best treatment option for maxillary interdental Class II furcation involvement depends on several factors, including:<br><br>Specific location and morphology of the furcation: Maxillary molars have three roots (mesiobuccal, distobuccal, and palatal), and access to the furcation can be challenging.<br><br>Amount of bone loss: Class II furcation involvement means some bone remains between the roots, but there is horizontal bone loss.<br><br>Presence of other periodontal issues: Overall periodontal health, inflammation, and plaque control play a role.<br><br>Patient factors: Overall health, compliance with oral hygiene, and smoking habits can influence treatment outcomes.<br><br>With that said, here's a breakdown of possible treatment options and their considerations:<br><br>1. Non-Surgical Therapy:	For maxillary interdental Class II furcation involvement, regenerative surgery is often the best treatment option1<br><br>This approach aims to reduce pocket depth and regenerate lost bone and tissue. Here are some common regenerative surgical options:<br><br>Flap Surgery: This involves lifting the gum tissue to access the roots and bone for thorough cleaning and reshaping.<br><br>Bone Grafts: Bone grafts can be used to fill in areas where bone has been lost, promoting new bone growth.<br><br>Guided Tissue Regeneration (GTR): This technique uses barrier membranes to encourage the growth of new bone and gum tissue while preventing the growth of unwanted tissues.<br><br>Consulting with a periodontist is essential to determine the most appropriate treatment plan based on the specific condition of the teeth and gums. Regular follow-ups and	In maxillary interdental Class II furcation involvement non-surgical instrumentation, OFD, periodontal regeneration, root separation or root resection may be considered.

	Bone Grafting: Consider using bone graft materials (autografts, allografts, xenografts, or alloplasts) in conjunction with GTR to promote bone regeneration within the furcation.<br><br>Root Resection: If the furcation involvement is severe and the tooth is restorable, root resection of the affected root may be an option, preserving the rest of the tooth.<br><br>3. Maintenance<br><br>Regular Follow-Up: Schedule frequent maintenance visits to monitor the periodontal health of the affected area and provide professional cleanings.<br><br>Patient Education: Emphasize effective home care techniques, including the use of interdental brushes or devices specifically designed for furcation access.<br><br>4. Consider Extraction<br><br>If the tooth shows a poor prognosis despite treatment, extraction may be necessary, followed by discussions about potential prosthetic replacements (like implants).<br><br>5. Multidisciplinary Approach<br><br>Collaborate with periodontists and restorative dentists to create a comprehensive treatment plan that addresses all aspects of the patient's oral health.<br><br>In summary, a combination of non-surgical and surgical interventions, tailored to the individual patient's needs, offers the best chances for successful management of maxillary interdental Class II furcation involvement.	to improve access for home care and to promote healing.<br><br>Gingival Grafting: If there is insufficient gum tissue coverage over the roots, a gingival graft may be performed to improve tissue thickness and prevent recession.<br><br>Regenerative Techniques: Bone grafts, bone substitutes, and growth factors can be used to encourage bone regeneration in the furcation area.<br><br>2. Non-Surgical Periodontal Therapy:<br><br>Deep Scaling and Root Planing: This involves removing plaque and tartar from the root surfaces and smoothing them to promote healing.<br><br>Antibiotic Therapy: In some cases, antibiotics may be prescribed to help control infection and promote healing.<br><br>3. Extraction:<br><br>If the tooth is severely damaged or cannot be restored, extraction may be necessary.<br><br>The most appropriate treatment option will be determined by a periodontist after a thorough examination of your mouth and medical history. It is important to discuss the risks, benefits, and potential outcomes of each option with your dentist to make an informed decision.	Scaling and root planing: This is always the first step to remove plaque and calculus, reduce inflammation, and create a favorable environment for healing. However, it alone is unlikely to regenerate lost bone in a Class II furcation.<br><br>2. Surgical Therapy:<br><br>Open flap debridement (OFD): This involves surgically accessing the furcation area, cleaning it thoroughly, and attempting to reduce the depth of the furcation. It may be combined with other techniques.<br><br>Guided tissue regeneration (GTR): A barrier membrane is placed to encourage regeneration of periodontal tissues (bone, cementum, and periodontal ligament). GTR can be more effective than OFD alone in Class II furcations, especially when combined with bone grafts or other biomaterials.<br><br>Bone grafting: Various bone grafts can be used to fill the furcation defect and promote bone regeneration. This is often used in conjunction with GTR.<br><br>Enamel matrix derivative (EMD): This protein gel can be applied to the root surface to promote regeneration. It may be used with GTR or OFD.<br><br>3. Resective Therapy:<br><br>Root resection/amputation: Involves removing one or more roots of the tooth. This can be an option if the furcation involvement is severe or access for regeneration is difficult.	maintenance are crucial for the success of the treatment.	

			This would typically be followed by restoration with a crown.<br><br>Tunneling: This creates a channel through the furcation to improve access for cleaning. It may be considered in specific cases.<br><br>4. Extraction:<br><br>If the tooth is deemed hopeless due to extensive bone loss, mobility, or other factors, extraction may be the best option. This would be followed by replacement options like a bridge or implant.<br><br>Important Notes:<br><br>Cone-beam computed tomography (CBCT) scans can be very helpful in assessing the furcation involvement and planning treatment.<br><br>Long-term success of any treatment depends heavily on meticulous oral hygiene and regular maintenance visits.<br><br>A periodontist is a dental specialist with expertise in treating periodontal disease and furcation involvement. It's essential to consult with a periodontist to determine the best treatment plan for your specific situation.		
**What is the best treatment option for maxillary Class III furcation involvement?**	Managing maxillary Class III furcation involvement requires a comprehensive approach due to the complexity of the condition. The best treatment options typically include:<br><br>1. Assessment<br><br>Clinical and Radiographic Evaluation: Assess the extent of furcation	Maxillary Class III furcation involvement is a severe periodontal condition where a deep pocket extends through the root of a tooth, making it difficult to clean and maintain. The best treatment option often depends on the specific circumstances of each case. Here are some common approaches:	Maxillary Class III furcation involvement presents a significant challenge, as it means there's a through-and-through communication between the roots with complete loss of bone within the furcation. This makes cleaning extremely difficult and increases the risk of periodontal disease progression.	For maxillary Class III furcation involvement, regenerative surgery is often the best treatment option<br><br>This approach aims to reduce pocket depth and regenerate lost bone and tissue	In maxillary Class III and multiple Class II furcation involvement in the same tooth nonsurgical instrumentation, OFD, tunneling, root separation or root resection may be considered.

	involvement and surrounding periodontal health.<br><br>2. Non-Surgical Therapy<br><br>Scaling and Root Planing (SRP): Perform thorough scaling and root planing to remove plaque and calculus from the tooth surface and the furcation area.<br><br>Antimicrobial Therapy: Consider local antimicrobials or systemic antibiotics if there is significant inflammation or infection.<br><br>3. Surgical Therapy<br><br>Flap Surgery: If deep pockets persist after non-surgical treatment, flap surgery may be indicated. This provides better access for debridement and allows for direct visualization of the furcation.<br><br>Guided Tissue Regeneration (GTR): Employ GTR techniques with appropriate membranes to promote regeneration of periodontal tissues and bone in the furcation area.<br><br>Bone Grafting: Consider using bone graft materials (autografts, allografts, xenografts, or alloplasts) to fill the furcation defect and support bone regeneration.<br><br>Root Resection: If the furcation involvement is significant and the tooth can be saved, root resection may be a viable option, which involves removing the affected root while preserving the rest of the tooth.<br><br>4. Maintenance<br><br>Regular Monitoring: Schedule frequent follow-up visits to assess periodontal health and provide maintenance cleanings.	1. Guided Tissue Regeneration (GTR)<br><br>Procedure: This involves using a barrier membrane to protect a bone graft from the surrounding tissues. This allows for the growth of new bone and periodontal tissue in the furcation defect.<br><br>Success: GTR has shown promising results in treating Class III furcation involvement, especially when combined with bone grafts.<br><br>2. Root Amputation<br><br>Procedure: This involves removing one or more roots of a multi-rooted tooth. If one root is severely compromised, it can be removed, leaving the remaining roots intact.<br><br>Suitability: Root amputation is often considered when the remaining roots have adequate bone support and the tooth can still function effectively.<br><br>3. Extraction<br><br>When: In severe cases where the tooth is non-restorable or the patient's overall oral health is compromised, extraction may be the most appropriate option.<br><br>Factors to Consider:<br><br>Tooth Vitality: If the tooth is non-vital (dead), it may be more difficult to achieve successful regeneration.<br><br>Bone Quality and Quantity: The amount and quality of bone surrounding the furcation defect can influence treatment outcomes.<br><br>Patient's Overall Health: The patient's general health and commitment to oral hygiene can impact the success of treatment.	Treatment options for maxillary Class III furcation involvement are more limited and often less predictable compared to Class II. Here's a breakdown of the possibilities:<br><br>1. Non-surgical therapy:<br><br>Scaling and root planing: While important for overall periodontal health, it won't resolve a Class III furcation defect on its own. It can, however, help improve the situation before surgical intervention.<br><br>2. Surgical therapy:<br><br>Guided tissue regeneration (GTR): GTR can be attempted, but its success rate is lower in Class III furcations due to the lack of supporting bone. It often requires careful case selection and may be combined with bone grafts and biomaterials.<br><br>Bone grafting: Bone grafting alone is unlikely to be successful in a Class III furcation, but it can be used in conjunction with GTR to try to promote some bone fill.<br><br>Enamel matrix derivative (EMD): Similar to GTR, EMD may have some benefit when used in combination with other techniques.<br><br>3. Resective therapy:<br><br>Root resection/amputation: This is often a more viable option for Class III furcations, especially if one or two	Here are some common regenerative surgical options:<br><br>Flap Surgery: This involves lifting the gum tissue to access the roots and bone for thorough cleaning and reshaping<br><br>Bone Grafts: Bone grafts can be used to fill in areas where bone has been lost, promoting new bone growth<br><br>Guided Tissue Regeneration (GTR): This technique uses barrier membranes to encourage the growth of new bone and gum tissue while preventing the growth of unwanted tissues<br><br>Consulting with a periodontist is essential to determine the most appropriate treatment plan based on the specific condition of the teeth and gums.<br><br>Regular follow-ups and maintenance are crucial for the success of the treatment	

	Patient Education: Educate the patient on proper oral hygiene techniques, particularly for furcation areas, using interdental brushes or other aids.<br><br>5. Consider Extraction<br><br>If the prognosis remains poor despite treatment efforts, extraction may be necessary. Discuss potential replacement options, such as implants or bridges.<br><br>6. Multidisciplinary Collaboration<br><br>Work with periodontists and restorative dentists to create a comprehensive treatment plan, ensuring all aspects of the patient's oral health are addressed.<br><br>In summary, a tailored combination of non-surgical and surgical treatments is essential for effectively managing maxillary Class III furcation involvement, aiming to preserve the tooth and restore periodontal health.	It's crucial to consult with a periodontist to determine the most suitable treatment option based on your individual situation. They will assess the severity of the furcation involvement, evaluate the surrounding tissues, and discuss your treatment goals to provide a personalized plan.	roots are compromised while others have adequate bone support. It simplifies the anatomy and allows for better cleaning. This would be followed by a crown.<br><br>Tunneling: While technically possible, tunneling is less ideal for Class III furcations as it leaves a significant open space that's difficult to maintain.<br><br>4. Extraction:<br><br>In many cases, extraction is the most predictable and practical solution for a maxillary Class III furcation, especially if the tooth has other issues like mobility, extensive bone loss, or root caries. It can be followed by replacement options like a bridge or implant.<br><br>Key considerations:<br><br>Complexity of maxillary molars: The anatomy of maxillary molars with their three roots and the curvature of the palatal root can make access and treatment more challenging.<br><br>Patient factors: Overall health, oral hygiene, and compliance are crucial for the success of any treatment.<br><br>Realistic expectations: Regenerating bone in a Class III furcation is difficult. Treatment often focuses on slowing disease progression, improving cleansibility, and preserving the tooth for as long as possible.<br><br>Periodontist consultation: It is crucial to consult with a periodontist to have a thorough evaluation, including CBCT scans if needed, and discuss the best treatment options for your specific situation.		

			Remember, I am an AI and cannot provide medical advice. Always consult with a qualified dental professional for diagnosis and treatment.		
**What is the best treatment option for mandibular Class III furcation involvement?**	Managing mandibular Class III furcation involvement requires careful planning and a combination of treatment modalities to effectively address the condition. Here are the best treatment options:<br><br>1. Assessment<br><br>Clinical Examination: Evaluate the extent of furcation involvement and assess periodontal health.<br><br>Radiographic Evaluation: Use radiographs to assess bone loss and the extent of the furcation defect.<br><br>2. Non-Surgical Therapy<br><br>Scaling and Root Planing (SRP): Conduct thorough SRP to remove plaque, calculus, and biofilm from the root surfaces and furcation area.<br><br>Antimicrobial Therapy: Consider local delivery of antimicrobials (e.g., chlorhexidine) or systemic antibiotics if there is significant inflammation or infection.<br><br>3. Surgical Therapy<br><br>Flap Surgery: If deep pockets persist after non-surgical treatment, flap surgery can provide better access to the furcation area for thorough debridement.<br><br>Guided Tissue Regeneration (GTR): Employ GTR techniques to promote the regeneration of periodontal tissues and bone in the furcation area, using	Mandibular Class III furcation involvement is a severe periodontal condition where a deep pocket extends through the root of a mandibular tooth, making it difficult to clean and maintain. The best treatment option often depends on the specific circumstances of each case. Here are some common approaches:<br><br>1. Guided Tissue Regeneration (GTR)<br><br>Procedure: This involves using a barrier membrane to protect a bone graft from the surrounding tissues. This allows for the growth of new bone and periodontal tissue in the furcation defect.<br><br>Success: GTR has shown promising results in treating Class III furcation involvement, especially when combined with bone grafts.<br><br>2. Root Amputation<br><br>Procedure: This involves removing one or more roots of a multi-rooted tooth. If one root is severely compromised, it can be removed, leaving the remaining roots intact.<br><br>Suitability: Root amputation is often considered when the remaining roots have adequate bone support and the tooth can still function effectively.<br><br>3. Extraction<br><br>When: In severe cases where the tooth is non-restorable or the patient's overall oral health is compromised, extraction may be the most appropriate option.	The best treatment option for mandibular Class III furcation involvement depends on several factors, including:<br><br>Specifics of the furcation involvement: How deep and wide is it? What's the overall periodontal health status?<br><br>Tooth's strategic importance: Is it vital for maintaining function and occlusion?<br><br>Patient's overall health and commitment to oral hygiene: Good oral hygiene is crucial for successful treatment.<br><br>Restorability of the tooth: Can the tooth be adequately restored after periodontal treatment?<br><br>With that said, here's a breakdown of possible treatment options and their considerations:<br><br>1. Non-surgical Treatment:<br><br>Scaling and root planing: This is the initial step for any periodontal treatment, aiming to remove plaque and tartar from the furcation area. However, it's rarely sufficient for Class III furcation involvement alone.<br><br>2. Surgical Treatment:<br><br>Open flap debridement: This allows for better access to clean the furcation, but may result in significant tissue recession and aesthetic concerns.	For mandibular Class III furcation involvement, regenerative surgery is often the best treatment option. This approach aims to reduce pocket depth and regenerate lost bone and tissue. Here are some common regenerative surgical options:<br><br>Flap Surgery: This involves lifting the gum tissue to access the roots and bone for thorough cleaning and reshaping.<br><br>Bone Grafts: Bone grafts can be used to fill in areas where bone has been lost, promoting new bone growth.<br><br>Guided Tissue Regeneration (GTR): This technique uses barrier membranes to encourage the growth of new bone and gum tissue while preventing the growth of unwanted tissues.<br><br>Consulting with a periodontist is essential to determine the most appropriate treatment plan based on the specific condition of the teeth and gums. Regular follow-ups and maintenance are crucial for the success of the treatment.	In mandibular Class III and multiple Class II furcation involvement in the same tooth nonsurgical instrumentation, OFD, tunneling, root separation or root resection may be considered.

	resorbable or non-resorbable membranes.<br><br>Bone Grafting: Use bone graft materials (autografts, allografts, xenografts, or alloplasts) to fill the furcation defect and support bone regeneration.<br><br>Root Resection: In cases where the furcation involvement is significant and the tooth is restorable, root resection may be a viable option, which involves removing the affected root while preserving the tooth.<br><br>4. Maintenance<br><br>Regular Follow-Up: Schedule regular recall visits to monitor periodontal health and provide maintenance cleanings.<br><br>Patient Education: Educate the patient on effective oral hygiene practices, especially for maintaining cleanliness in furcation areas, using tools like interdental brushes or special cleaning aids.<br><br>5. Consider Extraction<br><br>If the tooth has a poor prognosis despite treatment efforts, extraction may be necessary. Discuss potential replacement options, such as dental implants or bridges.<br><br>6. Multidisciplinary Approach<br><br>Collaborate with periodontists and restorative dentists to develop a comprehensive treatment plan that addresses all aspects of the patient's periodontal health.<br><br>In summary, a combination of non-surgical and surgical interventions tailored to the individual case is key to effectively managing mandibular Class III furcation involvement, aiming to	Factors to Consider:<br><br>Tooth Vitality: If the tooth is non-vital (dead), it may be more difficult to achieve successful regeneration.<br><br>Bone Quality and Quantity: The amount and quality of bone surrounding the furcation defect can influence treatment outcomes.<br><br>Patient's Overall Health: The patient's general health and commitment to oral hygiene can impact the success of treatment.<br><br>It's crucial to consult with a periodontist to determine the most suitable treatment option based on your individual situation. They will assess the severity of the furcation involvement, evaluate the surrounding tissues, and discuss your treatment goals to provide a personalized plan.	Guided tissue regeneration (GTR): Involves placing a barrier membrane to encourage regeneration of periodontal tissues. It can be combined with bone grafts for better results. This has shown promise in some cases, but long-term success is highly dependent on patient factors.<br><br>Furcation plasty: Reshaping the tooth roots to make the furcation area more accessible for cleaning. This is more common for maxillary molars.<br><br>Tunneling: Creating a tunnel through the furcation to allow access for cleaning. This is often considered for mandibular molars with Class III involvement. Success depends heavily on patient hygiene.<br><br>Extraction: In some cases, especially when other treatment options are unlikely to succeed or the tooth is not strategically important, extraction may be the most predictable solution. This is often followed by an implant or bridge.<br><br>3. Advanced Therapies:<br><br>Enamel matrix derivative (EMD): This involves applying a gel containing proteins found in developing teeth to stimulate tissue regeneration.<br><br>Platelet-rich fibrin (PRF): Utilizes a concentration of the patient's own growth factors to promote healing. A recent case report showed promising results combining PRF with bone grafts for treating Class III furcations.<br><br>Important Considerations:<br><br>Long-term prognosis: Even with successful treatment, teeth with Class III furcation involvement require meticulous oral hygiene and regular		

	preserve the tooth and restore periodontal health.		monitoring to prevent recurrence of periodontal disease.<br><br>Patient education: Thorough patient education about their condition, treatment options, and the importance of oral hygiene is critical for successful outcomes.<br><br>It's crucial to consult with a qualified periodontist to determine the best treatment option for your specific situation. They can perform a thorough examination, evaluate your overall health and oral hygiene, and discuss the risks and benefits of each treatment option.		
**Does adjunctive use of local drugs to subgingival instrumentation improve the clinical outcomes of furcation involvement?**	Yes, the adjunctive use of local drugs to subgingival instrumentation can improve clinical outcomes in the management of furcation involvement. Here are some key points regarding this approach:<br><br>Benefits of Adjunctive Local Drug Therapy<br><br>Enhanced Antimicrobial Action: Local delivery of antimicrobials (e.g., chlorhexidine, minocycline, or doxycycline) directly targets the periodontal pathogens in the affected area, reducing bacterial load more effectively than SRP alone.<br><br>Reduced Inflammation: These agents can help decrease inflammation, promoting better healing of the periodontal tissues.<br><br>Improved Pocket Depth Reduction: Studies have shown that the combination of SRP with local antimicrobial agents often results in greater reductions in probing depth compared to SRP alone, especially in furcation areas.	Adjunctive Local Drugs for Furcation Involvement: A Review<br><br>Yes, adjunctive use of local drugs to subgingival instrumentation can improve the clinical outcomes of furcation involvement.<br><br>While scaling and root planing (SRP) remains the cornerstone of non-surgical periodontal therapy, including furcation involvement, adjunctive therapies can enhance treatment outcomes. Local drug delivery can target specific areas, reducing the potential for systemic side effects and improving compliance.<br><br>Common Adjunctive Therapies<br><br>Antibiotics: Tetracycline, minocycline, and metronidazole are commonly used. They can help reduce bacterial loads in the furcation area, promoting healing.<br><br>Antiseptics: Chlorhexidine and essential oils can be used to disinfect the furcation area and reduce inflammation.<br><br>Host modulation agents: Bisphosphonates and statins have shown promise in enhancing bone	While subgingival instrumentation (like scaling and root planing) is the cornerstone of treating furcation involvement, adjunctive use of local drugs can indeed improve clinical outcomes in some cases. However, the evidence is mixed, and the effectiveness varies depending on the specific drug used.<br><br>Here's a breakdown of what we know:<br><br>Potential Benefits of Local Drug Delivery:<br><br>Enhanced antimicrobial action: Local delivery of antimicrobials (like doxycycline, chlorhexidine, or metronidazole) can help to further reduce the bacterial load in the furcation area, improving the effectiveness of mechanical debridement.<br><br>Reduced inflammation: Some drugs have anti-inflammatory properties that can help to control inflammation and promote healing.<br><br>Stimulated tissue regeneration: Certain drugs, such as enamel matrix derivatives (EMD) and growth factors,	Yes, the adjunctive use of local drugs to subgingival instrumentation can improve clinical outcomes for furcation involvement. Studies have shown that supplementing mechanical debridement (such as scaling and root planing) with locally delivered antimicrobials can enhance treatment results. These local drugs help reduce bacterial biofilms in difficult-to-reach areas, such as furcations and deep pockets, leading to better clinical outcomes1	Superior clinical treatment outcomes were shown when alendronate, rosuvastatin, boric acid, simvastatin, and tetracycline (only at 3 months) were utilized in furcation defects in conjunction with SRP alone or SRP + placebo. Significant improvement was reported in radiographic bone defect depth and defect depth reduction when SRP was supplemented with alendronate, rosuvastatin, boric acid, and simvastatin.<br><br>The various local drugs used in treating class II mandibular furcation defects are effective in the prevention of bleeding on probing, bone resorption, gingival bleeding index and increase in the bone fill, and microbial deposit removal.

	Enhanced Clinical Attachment Levels: Adjunctive local therapies may lead to improved clinical attachment gain in patients with furcation involvement.<br><br>Sustained Effect: Local delivery systems can provide a sustained release of the drug over time, maintaining effective concentrations at the site of infection.<br><br>Considerations<br><br>Specificity of Drug Action: The choice of drug may depend on the specific bacterial profile of the patient and the type of furcation involvement.<br><br>Patient Compliance: Ensuring patients understand the importance of adjunctive therapies and maintaining good oral hygiene is critical for maximizing outcomes.<br><br>Individualized Treatment Plans: Treatment should be tailored based on the severity of furcation involvement, patient health, and response to prior treatments.<br><br>In conclusion, the adjunctive use of local drugs in conjunction with subgingival instrumentation can lead to improved clinical outcomes in patients with furcation involvement, making it a valuable addition to periodontal therapy.	regeneration and reducing inflammation.<br><br>Benefits of Adjunctive Therapy<br><br>Improved clinical attachment levels: Local drug delivery can help to reduce pocket depth and improve clinical attachment levels.<br><br>Enhanced bone regeneration: Certain drugs, such as bisphosphonates, can stimulate bone formation in furcation defects.<br><br>Reduced inflammation: Anti-inflammatory agents can help to reduce tissue inflammation and promote healing.<br><br>Improved patient compliance: Local drug delivery can be more convenient for patients than systemic medications.<br><br>It's important to note that the effectiveness of adjunctive therapy can vary depending on the specific drug, the severity of the furcation involvement, and other factors. Consultation with a dental professional is essential to determine the most appropriate treatment plan for individual cases.	can promote the regeneration of periodontal tissues, including bone.<br><br>Evidence from Studies:<br><br>Antimicrobials: Some studies have shown that local delivery of antimicrobials can lead to short-term improvements in probing depth and attachment levels in furcation defects. However, long-term benefits are less clear, and some studies have found no significant difference compared to subgingival instrumentation alone.<br><br>Host-modulating agents: Drugs like alendronate and simvastatin have shown promising results in improving clinical and radiographic outcomes in furcation defects. These agents work by modulating the host response to inflammation and promoting bone regeneration.<br><br>Other agents: Tetracycline fibers, minocycline microspheres, and chlorhexidine chips have also been studied, with varying degrees of success.<br><br>Factors to Consider:<br><br>Type and severity of furcation involvement: The effectiveness of local drug delivery may vary depending on the specific type and severity of the furcation defect.<br><br>Specific drug used: Different drugs have different mechanisms of action and levels of evidence supporting their use.<br><br>Patient compliance: The success of local drug delivery often depends on patient compliance with oral hygiene instructions and follow-up appointments.<br><br>Overall:		

			Adjunctive use of local drugs can be a valuable tool in the treatment of furcation involvement, but it's not a magic bullet.<br><br>The decision to use local drugs should be made on a case-by-case basis, considering the specific characteristics of the furcation defect, the patient's overall health and compliance, and the available evidence supporting the use of the specific drug.<br><br>It's important to discuss the potential benefits and risks of local drug delivery with your periodontist to determine if it's the right option for you.		
**Does adjunctive use of systemic antibiotics improve the clinical outcomes of furcation involvement?**	Yes, the adjunctive use of systemic antibiotics can improve clinical outcomes for furcation involvement in certain cases. Here are some important points regarding their use:<br><br>Benefits of Systemic Antibiotics<br><br>Broader Antimicrobial Coverage: Systemic antibiotics can target a wider range of periodontal pathogens, particularly in cases where specific bacterial profiles are not well understood.<br><br>Enhanced Clinical Outcomes: Studies have shown that systemic antibiotics, when used alongside scaling and root planing (SRP), can lead to greater reductions in probing depths and improvements in clinical attachment levels compared to SRP alone, particularly in more severe cases of furcation involvement.<br><br>Reduced Inflammation: Antibiotics can help reduce inflammation and support healing by controlling bacterial infections that contribute to periodontal disease.	Yes, systemic antibiotics can improve the clinical outcomes of furcation involvement.<br><br>While local drug delivery can target specific areas, systemic antibiotics offer a broader approach to combatting infection. They can help to reduce the overall bacterial load in the mouth, which can be beneficial in cases of severe or spreading infection.<br><br>Common Systemic Antibiotics<br><br>Tetracyclines: Doxycycline and minocycline are often used due to their ability to concentrate in gingival tissue.<br><br>Penicillins: Amoxicillin is a common choice, often combined with clavulanic acid (Augmentin) to enhance its effectiveness against beta-lactamase-producing bacteria.<br><br>Cephalosporins: Cefuroxime and cefixime are other options that can be effective against a wide range of bacteria.<br><br>Benefits of Systemic Antibiotics	While systemic antibiotics can be a helpful adjunct to periodontal therapy in certain cases, their use in treating furcation involvement specifically is somewhat controversial. Here's a breakdown of what we know:<br><br>Potential Benefits:<br><br>Reaching deep infections: Systemic antibiotics can penetrate deep into periodontal tissues, including furcation areas, where mechanical debridement alone may not be fully effective. This can help reduce the bacterial load and inflammation.<br><br>Improved healing: Some studies suggest that adjunctive antibiotics may lead to slightly better pocket reduction and attachment gain at furcation sites compared to scaling and root planing alone.<br><br>Limitations and Concerns:<br><br>Limited clinical significance: The improvements seen with antibiotics are often small and may not be clinically significant in the long term.	The adjunctive use of systemic antibiotics can improve clinical outcomes for furcation involvement, but it's not always necessary or recommended for every case. Systemic antibiotics are typically reserved for more severe or persistent cases of periodontal disease, where local treatments alone may not be sufficient.	**There is no benefit with the adjunctive use of systemic antibiotics**

	Synergistic Effects: In cases of aggressive periodontitis or when there is significant inflammation, systemic antibiotics may work synergistically with local treatments to enhance overall therapeutic effects.<br><br>Considerations<br><br>Appropriate Selection: The choice of antibiotic should be based on the specific clinical situation, including any known antibiotic sensitivities of the bacteria involved.<br><br>Patient Health: Consideration of the patient's medical history, potential drug interactions, and risk of antibiotic resistance is essential.<br><br>Compliance: Patient adherence to the antibiotic regimen is critical for achieving optimal outcomes.<br><br>Individualized Treatment: Systemic antibiotics should be considered as part of a comprehensive treatment plan that includes mechanical debridement and ongoing maintenance.<br><br>Conclusion<br><br>While adjunctive systemic antibiotics can improve clinical outcomes in managing furcation involvement, they should be used judiciously and tailored to individual patient needs. Combining them with other periodontal therapies, such as SRP, often yields the best results.	Reduced bacterial load: Systemic antibiotics can help to reduce the overall bacterial burden in the mouth, including in the furcation area.<br><br>Improved inflammation: Antibiotics can help to reduce inflammation, which can promote healing.<br><br>Enhanced clinical attachment levels: By combating infection, systemic antibiotics can contribute to improved clinical attachment levels.<br><br>Prevention of spreading infection: In cases of severe or spreading infection, systemic antibiotics can help to prevent the spread of bacteria to other areas of the mouth.<br><br>However, it's important to note that systemic antibiotics should be used judiciously. Overuse can contribute to antibiotic resistance. Consultation with a dental professional is essential to determine the appropriate course of treatment, including the need for systemic antibiotics.	Antibiotic resistance: Overuse of antibiotics can contribute to the development of antibiotic-resistant bacteria, making future infections harder to treat.<br><br>Side effects: Antibiotics can cause side effects like gastrointestinal upset, allergic reactions, and oral thrush.<br><br>Not a substitute for mechanical therapy: Systemic antibiotics should never be considered a replacement for thorough scaling and root planing, which is the cornerstone of furcation treatment.<br><br>Current Recommendations:<br><br>Case-by-case basis: The decision to use systemic antibiotics should be made on a case-by-case basis, considering factors like the severity of the furcation involvement, the patient's overall health, and the presence of other risk factors.<br><br>Specific situations: Antibiotics may be more beneficial in cases of severe furcation involvement, aggressive periodontitis, or when mechanical therapy alone is insufficient.<br><br>Combination therapy: Combining amoxicillin and metronidazole is a common regimen for periodontal infections.<br><br>Conclusion:<br><br>While adjunctive systemic antibiotics may offer some benefits in treating furcation involvement, their use should be carefully considered and reserved for specific situations. More research is needed to determine the optimal use of antibiotics in periodontal therapy and to identify patients who are most likely to benefit.		

			It's crucial to consult with a periodontist to determine the best course of treatment for your specific needs. They can assess your condition and recommend the most appropriate therapy, which may include a combination of mechanical debridement, local antimicrobial agents, and possibly systemic antibiotics.		
**What is the best imaging technique for assessing furcation defects?**	The best imaging techniques for assessing furcation defects in periodontal disease include:<br><br>1. Digital Radiography<br><br>Periapical Radiographs: These provide detailed images of specific teeth and their surrounding structures, allowing for assessment of bone levels and furcation involvement. However, their limitation is that they may not always show the extent of the furcation defect clearly.<br><br>2. Cone Beam Computed Tomography (CBCT)<br><br>3D Imaging: CBCT offers a three-dimensional view of the periodontal structures, providing detailed information about the extent of bone loss, furcation involvement, and the anatomy of the roots and furcation areas. This technique is particularly valuable for complex cases and pre-surgical planning.<br><br>3. Periodontal Probing<br><br>While not an imaging technique, clinical probing is essential for assessing the depth of furcation involvement. Combining probing data with imaging provides a comprehensive evaluation.<br><br>4. Ultrasonography	The best imaging technique for assessing furcation defects is cone beam computed tomography (CBCT). Here's why:<br><br>Superior accuracy: CBCT provides a 3D image of the tooth and surrounding bone, allowing for more precise measurement and visualization of furcation defects than traditional 2D radiographs.<br><br>Detailed information: CBCT can reveal the extent of bone loss, the presence of any periodontal pockets, and the root anatomy.<br><br>Improved treatment planning: CBCT helps dentists plan more targeted and effective treatments for furcation defects, including regenerative therapies and surgical procedures.<br><br>While traditional radiographs can still be helpful in some cases, CBCT offers a significant advantage in terms of accuracy and detail.	Cone-beam computed tomography (CBCT) is currently considered the best imaging technique for assessing furcation defects. Here's why:<br><br>3D visualization: CBCT provides detailed three-dimensional images of the tooth and surrounding bone, allowing for accurate assessment of the furcation defect's location, extent, and morphology. This is crucial for treatment planning and prognosis.<br><br>Accurate measurements: CBCT allows for precise measurements of furcation involvement, including vertical and horizontal bone loss. This information is essential for determining the appropriate treatment approach.<br><br>Detection of subtle defects: CBCT can detect subtle furcation involvements that may not be visible on traditional radiographs. This can lead to earlier diagnosis and intervention.<br><br>Evaluation of surrounding structures: CBCT allows visualization of adjacent teeth, roots, and anatomical structures, which can aid in treatment planning and avoid complications during surgery.<br><br>Comparison to other imaging techniques:	The best imaging technique for assessing furcation defects is Cone-Beam Computed Tomography (CBCT).<br><br>CBCT provides detailed 3D images of the teeth and surrounding structures, allowing for accurate assessment of furcation involvement<br><br>. It is more effective than traditional 2D radiographs (such as periapical or panoramic radiographs) in detecting and evaluating the extent of furcation defects	CBCT is the best imaging technique for assessing furcation defects

	Although less common, ultrasonography can be used to assess soft tissue changes and possibly provide information about the periodontal structures in real time.<br><br>Conclusion<br><br>CBCT is generally considered the best imaging technique for assessing furcation defects due to its ability to provide detailed, three-dimensional images of the anatomical structures involved. However, digital radiography still plays an important role in routine assessments, and a combination of imaging techniques may be used for comprehensive evaluation and treatment planning.		Traditional radiographs (periapical and bitewing): These 2D images offer limited information about furcation involvement due to superimposition of structures and distortion. They often underestimate the true extent of the defect.<br><br>Periodontal probing: While essential for clinical examination, probing alone can be subjective and influenced by factors like inflammation, root morphology, and operator technique.<br><br>Limitations of CBCT:<br><br>Radiation exposure: CBCT involves a higher radiation dose compared to traditional radiographs. However, newer CBCT units offer lower-dose protocols.<br><br>Cost: CBCT scans are generally more expensive than traditional radiographs.<br><br>Availability: CBCT scanners may not be readily available in all dental practices.<br><br>Conclusion:<br><br>Despite its limitations, CBCT offers significant advantages over other imaging techniques in assessing furcation defects. Its ability to provide detailed 3D images and accurate measurements makes it a valuable tool for diagnosis, treatment planning, and monitoring of periodontal disease.		
